# Diagnosis and treatment of a patient with mediastinal infection caused by *Emergomyces orientalis* and *Mycobacterium fortuitum*

**DOI:** 10.3389/fcimb.2026.1778930

**Published:** 2026-04-24

**Authors:** Liang Guo, Xiaoli Luo, Menglin Luo, Mingzhou Zhang, Bin Wang, You Fu, Xuemei Wu, Yue Yu, Li Bai, Zhi Xu

**Affiliations:** 1 Institute of Respiratory Disease, Xinqiao Hospital, Second Affiliated Hospital, Army Medical University, Chongqing, China; 2 Institute of Pharmacy, Xinqiao Hospital, Second Affiliated Hospital, Army Medical University, Chongqing, China; 3 Institute of Pathology, Xinqiao Hospital, Second Affiliated Hospital, Army Medical University, Chongqing, China

**Keywords:** *Emergomyces orientalis* (*E. orientalis*), endobronchial ultrasound (EBUS), mediastinal mixed infection, metagenomic next-generation sequencing (mNGS), *Mycobacterium fortuitum (M. fortuitum)*

## Abstract

**Background:**

Emergomycosis, an emerging dimorphic fungal infection caused by *Emergomyces* species, primarily affects immunocompromised individuals. *Emergomyces orientalis* has been reported in China, including rare cases in immunocompetent individuals. Diagnosis remains challenging due to the lack of typical clinical manifestations and radiological features. Co-infection with other pathogens further complicates management, with no prior global reports of concurrent *E. orientalis* and non-tuberculous mycobacterial (NTM) infections.

**Case presentation:**

A 21-year-old immunocompetent woman with occupational exposure to soil presented with cough, fever, and a mediastinal mass on chest CT. The initial biopsy specimens revealed granulomatous inflammation and yeast-like fungi. Metagenomic next-generation sequencing (mNGS) of endobronchial ultrasound (EBUS)-guided specimens confirmed *E. orientalis* (40 reads). Liposomal amphotericin B induction therapy initially relieved the symptoms. However, recurrence prompted repeat mNGS, which revealed elevated *Mycobacterium fortuitum* loads (791 reads). Combined with the patient’s history of soil exposure, a diagnosis of mediastinal *E. orientalis* with *M. fortuitum* co-infection was established based on the clinical presentation, the chest CT findings, histopathological observations of yeast-like fungi, the mNGS results, and the therapeutic response. Following confirmation of the co-infection, tailored adjustments to the antimicrobial regimen led to successful clinical management.

**Conclusion:**

To the best of our knowledge, this is the first study in which *E. orientalis* and *M. fortuitum* were documented to coexist in the mediastinum. The dual pathogens were identified through a combination of EBUS-guided biopsy and mNGS. Accurate pathogen identification followed by tailored, pathogen-directed therapy is essential for the effective management of an *E. orientalis* and *M. fortuitum* mixed infection.

## Introduction

*Emergomyces* is a newly recognized fungal genus of clinical significance. In nature, it exists as a saprophytic mold; however, upon infecting a mammalian host, it undergoes a critical morphological shift into a yeast-like form (Schwartz et al., 2019). Immunocompromised individuals, such as those with HIV, solid organ transplants, or prolonged immunosuppressive therapy, are at the highest risk of the disease (Govender and Grayson, 2019; Schwartz et al., 2019). However, reports of *Emergomyces orientalis* infection in immunocompetent individuals have also emerged in recent years (Wang et al., 2017; He et al., 2021; Jiang et al., 2024; Luo et al., 2024).

*E. orientalis* infection can involve multiple sites, such as the skin, respiratory system, hematopoietic system, central nervous system, digestive system, and reproductive system. In patients with respiratory involvement, imaging may reveal diffuse or focal reticulonodular infiltrates, consolidation, lobar atelectasis, pleural effusion, and hilar lymphadenopathy. Cases presenting with mediastinal or hilar lymphadenopathy require differentiation from other conditions, including lymphoma, sarcoidosis, tuberculosis, non-tuberculous mycobacterial (NTM) infection, and various immune-related diseases. Due to the nonspecific imaging features, diagnosis is often challenging and typically relies on combining clinical features, radiological findings (especially chest CT), microbiological culture, histopathology, and molecular techniques such as PCR or sequencing (Thompson et al., 2021).

We report on a case of mediastinal co-infection with *E. orientalis* and *Mycobacterium fortuitum*—a presentation not previously documented in the literature. The diagnosis in this patient was achieved through repeated endobronchial ultrasound (EBUS)-guided mediastinal biopsies, histopathology, metagenomic next-generation sequencing (mNGS), and assessment of the therapeutic response. Given that there are currently no international or domestic guidelines for the treatment of *E. orientalis* infection or its co-infection, drug selection for the treatment of *E. orientalis* co-infection with *M. fortuitum* is primarily based on literature reports regarding *E. orientalis* and the Infectious Diseases Society of America (IDSA) guidelines for NTM therapy, with personalized adjustments made according to the patient’s clinical condition. This case enhances understanding of dual infection with two rare pathogens and provides a reference for the diagnosis and management of similar cases.

## Case presentation

A 21-year-old woman with no underlying medical conditions presented to our clinic on April 20, 2025, for a mild cough and fever. At 3 weeks prior, she visited another hospital for chest computed tomography (CT), which revealed middle and upper mediastinal masses. Levofloxacin was administered orally; however, the symptoms did not improve significantly. She was hospitalized on April 20, 2025. The patient had no history of disease; however, she worked outdoors, such as training, running, and mountaineering, which involved exposure to and inhalation of large amounts of dust 3 months before the onset of the disease.

**Figure 1:**
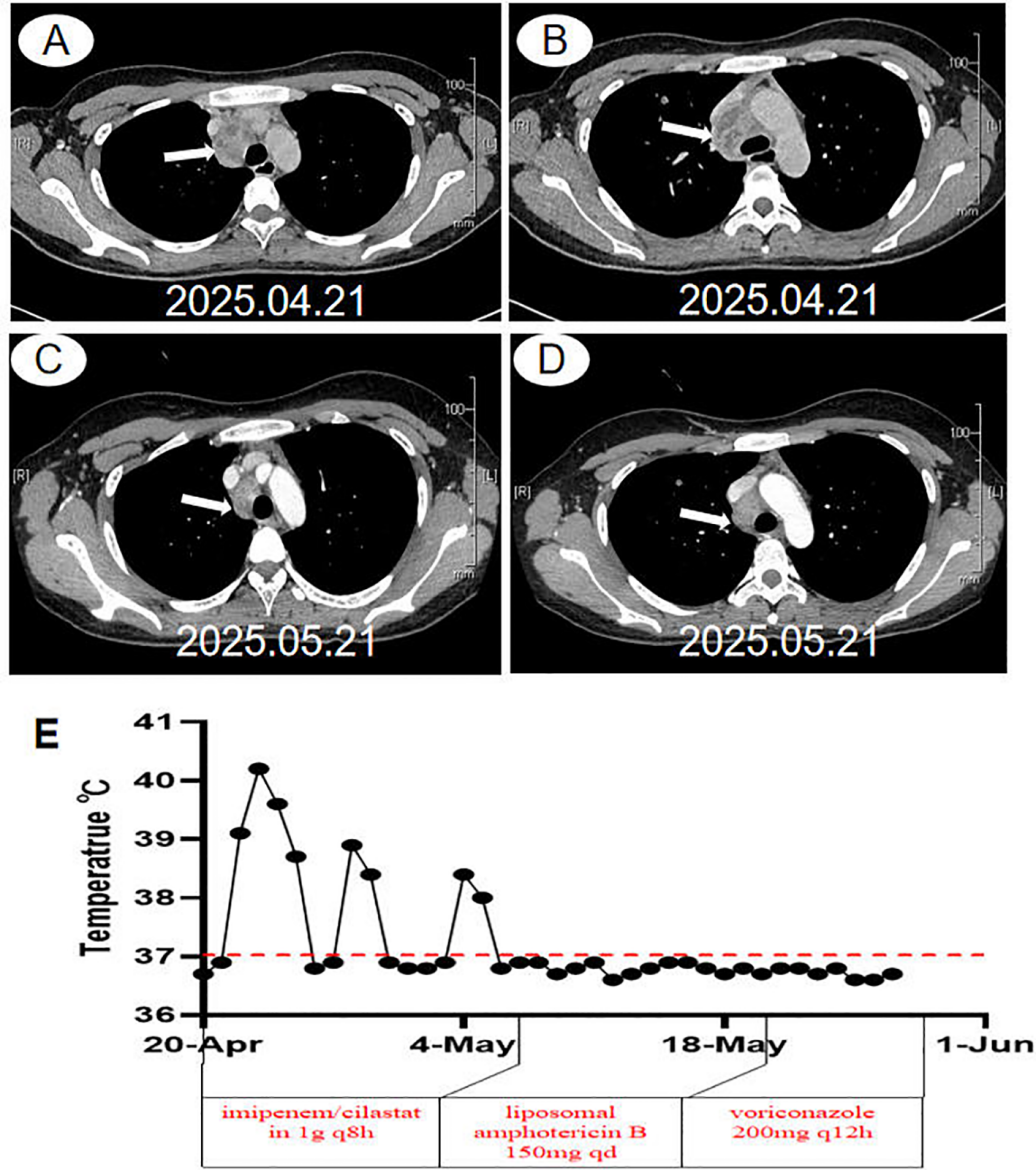
Chest computed tomography images and the changes in temperature and drug use of the patient at the first visit. **(A, B)** Initial contrast-enhanced chest CT revealing a 4.0-cm × 3.8-cm mass in the upper–middle mediastinum (*white arrow*). The mass demonstrates heterogeneous internal density and heterogeneous enhancement, with associated compression of the superior vena cava. **(C, D)** Follow-up chest CT performed on the 14th day of treatment showing a marked reduction in the size of the mediastinal lesion, which measures approximately 2.8 cm × 2.0 cm (*white arrow*). **(E)** Changes in temperature and drug use of the patient at the first visit.

No cutaneous rashes, superficial lymphadenopathy, hepatosplenomegaly, or other abnormal findings were noted on full physical examination. At the initial workup, as shown in **Table 1**, her HIV antibody test was negative, and the T-cell subpopulation analysis revealed a total T-cell count of 673 cells/µl, a CD4^+^ T-lymphocyte count of 309 cells/µl, and a CD8^+^ T-lymphocyte count of 273 cells/µl. The C-reactive protein (CRP) concentration was 42.6 mg/L. The white blood cell (WBC) count was 8.37 × 10^9^/L. The hemoglobin (HGB) concentration was 124 g/L, and the platelet (PLT) count was 266 × 10^9^/L. The neutrophil count was 5.49 × 10^9^/L, while the lymphocyte count was 2.09 × 10^9^/L. Procalcitonin, aspartate aminotransferase (AST), alanine aminotransferase (ALT), bilirubin, and the renal function tests were within normal limits. The prealbumin concentration was 115 mg/L, and the albumin concentration was 36.8 g/L. Serologic testing for fungal pathogens revealed negative results for the *Aspergillus* galactomannan assay and the cryptococcal capsular antigen assay. The 1,3-beta-d-glucan concentration was 81 pg/ml, and the T-cell spot test for *Mycobacterium tuberculosis* infection was negative. Autoimmune serology, including the antinuclear antibody (ANA) profile, anti-neutrophil cytoplasmic antibodies (ANCAs), and anti-cardiolipin antibodies (ACAs), was uniformly negative. Chest enhancement CT revealed a 4.0-cm × 3.8-cm mass in the upper–middle mediastinum. The mass exhibited heterogeneous internal density and showed heterogeneous enhancement on the contrast scan, with associated compression of the superior vena cava (**Figures 1A, B**).

**Table 1:** Laboratory parameters at the initial workup.

Laboratory parameters	Value	Normal range
C-reactive protein (mg/L)	42.6	0–8
White blood cell (×10^−9^/L)	8.37	3.50–9.50
Neutrophil count (×10^−9^/L)	5.49	1.8–6.3
Lymphocyte count (×10^−9^/L)	2.09	1.1–3.2
Hemoglobin (g/L)	124	115–150
Platelet (×10^−9^/L)	266	100–300
Procalcitonin (mg/ml)	<0.05	0–0.1
Aspartate aminotransferase (IU/L)	11	13–35
Alanine aminotransferase (IU/L)	5	7–40
Creatinine (μmol/L)	59	41–81
Bilirubin (μmol/L)	8.4	0–21
Albumin (g/L)	36.8	40–55
Prealbumin (mg/L)	115	180–350
Total T-cell count (cells/µl)	673	955–2,860
CD4^+^ T-lymphocyte count (cells/µl)	309	500–1,440
CD8^+^ T-lymphocyte count (cells/µl)	273	238–1,250
Serologic *Aspergillus* galactomannan	Negative	Negative
Serologic 1,3-beta-d-glucan test (pg/ml)	81	0–60
Cryptococcal capsular antigen	Negative	Negative
T-cell spot test	Negative	Negative
HIV antibody	Negative	Negative
Autoimmune serology (ANA, ANCA, ACA)	Negative	Negative

ANA, antinuclear antibody; ANCA, antineutrophil cytoplasmic antibodies; ACA, anticardiolipin antibodies.

**Figure 2:**
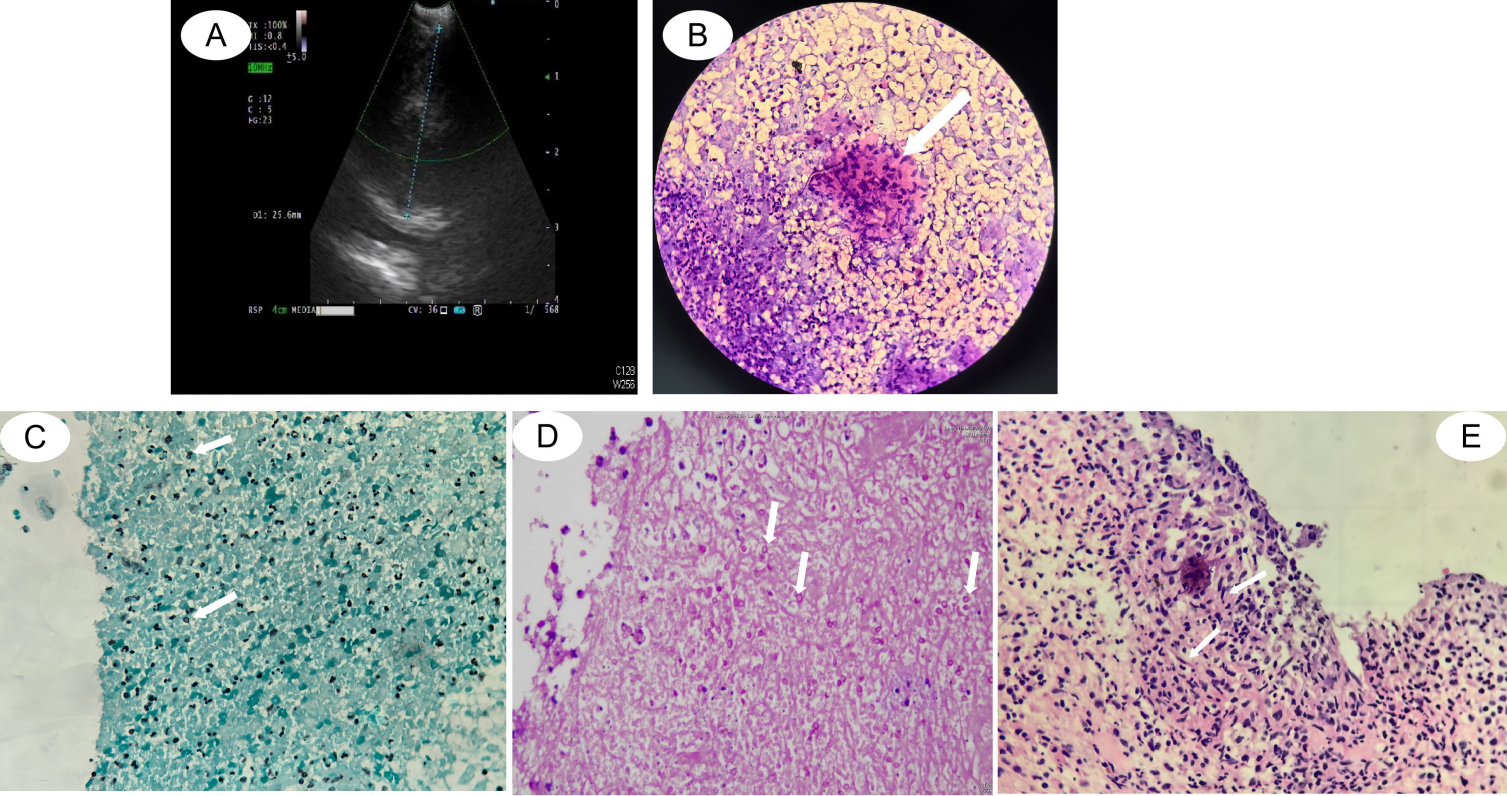
Tissue pathology images of the mediastinal biopsy tissue. **(A)** Endobronchial ultrasound (EBUS)-guided biopsy of the mediastinal lesions. **(B)** An EBUS brushing smear revealing granulomatous inflammation in the first stage (×40). **(C)** Gomori methenamine silver (GMS) staining of an EBUS–transbronchial needle aspiration (TBNA) specimen showing yeast-like fungi in the first stage (×40). **(D)** Periodic acid–Schiff (PAS) staining of the EBUS-TBNA specimen showing yeast-like fungi in the first stage (×40). **(E)** EBUS cryobiopsy specimen showing epithelioid cells in the second stage (×40).

To elucidate the disease etiology, EBUS brushing, EBUS-guided transbronchial needle aspiration (EBUS-TBNA), and cryobiopsy of the mediastinal lesion were performed (**Figure 2A**). Unfortunately, at 8 h post-biopsy, the patient developed a fever, reaching a maximum body temperature of 40.4°C accompanied by rigors. The temperature changes are shown in **Figure 1E**. Concurrently, the CRP levels increased, and pancytopenia developed (WBC count nadir, 1.57 × 10^9^/L). The transaminase levels increased progressively [ALT = 496 U/L, AST = 736 U/L, gamma-glutamyl transferase (γ-GGT) = 413 U/L, and alkaline phosphatase (ALP) = 306 U/L]. The bone marrow aspirate revealed no evidence of hematologic malignancy or hypercellular marrow. Positron emission tomography–computed tomography (PET-CT) demonstrated enlargement of the 2R and 4R lymph node stations [measuring 3.06 cm × 2.19 cm, with a maximum standardized uptake value (SUV_max_) of 6.56], which was radiologically interpreted as infectious rather than malignant (**Supplementary Figures 1A, B**). Empirical antibiotic therapy with imipenem/cilastatin (1 g intravenously every 8 h, q8h) was initiated promptly; however, intermittent fever persisted.

After several days, the pathological results were as follows: epithelioid-like cells were identified in the EBUS brush smear (**Figure 2B**), and acute and chronic inflammation accompanied by fibrous tissue proliferation was detected in the cryobiopsy specimens. Abundant necrotic substances and neutrophil aggregation were observed, and the presence of yeast-like fungi in localized necrotic tissue was detected in the EBUS-TBNA sample, indicating fungal infection. Gomori methenamine silver (GMS) staining and periodic acid–Schiff (PAS) staining were positive (**Figures 2C, D**). mNGS of the EBUS-TBNA specimens revealed the presence of *E. orientalis* (40 reads) and *M. fortuitum* (six reads). On the basis of the clinical manifestations and the pathological and mNGS results, the patient was considered to have *E. orientalis* infection in the mediastinum. However, given the low NTM sequence count and the absence of typical imaging features, the possibility that *M. fortuitum* originated from water contamination during the tissue sample process steps, such as washing and dehydration, cannot be ruled out. It was therefore considered a contaminant. With respect to the treatment of *E. orientalis*, there is a lack of recommendations in both domestic and international guidelines and consensus documents, in particular for immunocompetent individuals. In accordance with the literature reports, a regimen of liposomal amphotericin B followed by azole is recommended. Therefore, the patient was intravenously administered liposomal amphotericin B at a dosage of 150 mg once daily for antifungal therapy. Her body temperature returned to normal 2 days after the initiation of treatment. A follow-up chest CT scan performed on the 14th day of treatment revealed a marked reduction in the mediastinal lesions compared with prior imaging, with the lesion measuring approximately 2.8 cm × 2.0 cm. The mass exhibited heterogeneous internal density and showed heterogeneous enhancement on the contrast scan (**Figures 1C, D**). The changes in temperature are shown in **Figure 1E**. The patient was discharged in an improved condition and continued to receive treatment on an outpatient basis. After discharge, the antifungal regimen was switched to oral voriconazole (200 mg orally every 12 h).

**Figure 3:**
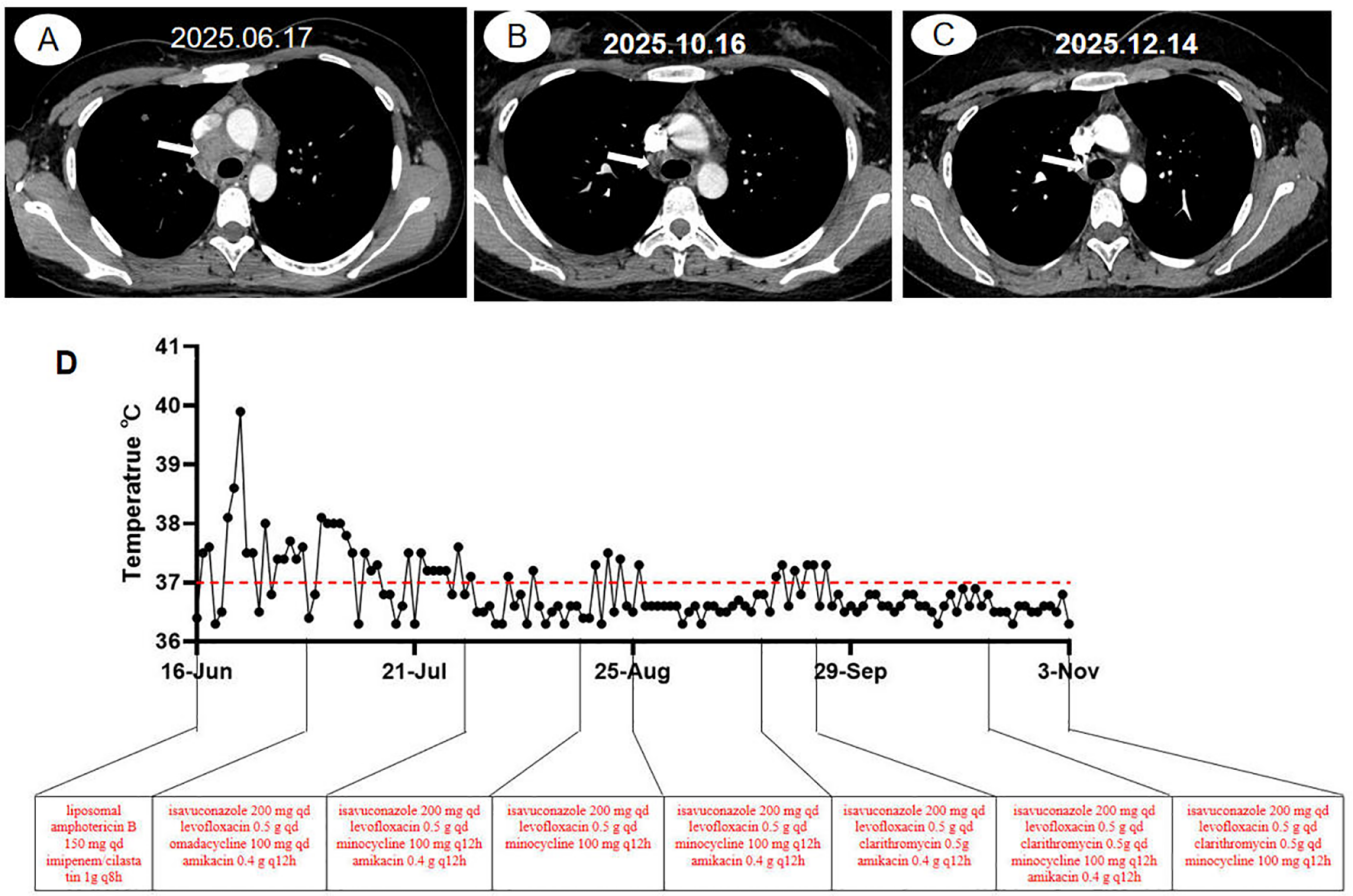
Chest computed tomography images and changes in temperature and drug use of the patient during the second hospitalization. **(A)** Contrast-enhanced chest CT 2 weeks after discharge from the first phase of treatment showing interval enlargement of the mediastinal lesion to approximately 3.6 cm × 3.3 cm (*white arrow*). The mass demonstrates heterogeneous internal density and heterogeneous enhancement. **(B)** At 1 month after the initiation of the second-phase anti-infective therapy, the follow-up CT demonstrates a significant reduction in the lesion size, now measuring 1.8 cm × 1.7 cm (*white arrow*). **(C)** After the modified treatment regimen, the mediastinal lesions reduced to a residual 1.3-cm × 1.1-cm nub (*white arrow*). **(D)** Changes in temperature and drug use of the patient during the second hospitalization.

After 2 weeks, the patient presented with fever recurrence, weight loss, and enlargement of the mediastinal lesions, measuring approximately 3.6 cm × 3.3 cm on contrast-enhanced scan, with heterogeneous internal density (**Figure 3A**) and with increased uptake (SUV_max_ = 6.72) (**Supplementary Figures 1C, D**) relative to those in prior assessments. The changes in temperature are shown in **Figure 3D**. The differential diagnosis included possible voriconazole ineffectiveness, concurrent co-infection, or lymphoma given the recurrence of the patient’s symptoms. Following empirical withdrawal of voriconazole and initiation of liposomal amphotericin B (150 mg intravenously qd) and imipenem/cilastatin (1 g intravenously q8h), the patient’s fever and mediastinal lesions remained uncontrolled. However, the G-test result decreased to 33 pg/ml. To further clarify the etiology, repeated EBUS brushing, EBUS-guided cryobiopsy, and TBNA of the mediastinal tissue yielded the following pathological findings: the presence of epithelioid-like cells identified in the EBUS brushing smear and the presence of epithelioid-like cells with hemorrhage and inflammatory cell infiltration detected in the cryobiopsy specimen (**Figure 2E**). Abundant necrotic material with neutrophilic aggregates was observed in the EBUS-TBNA sample. TB-DNA was negative. mNGS of the mediastinal needle biopsy specimens revealed *M. fortuitum* (791 reads) and *Legionella pneumophila* (58 reads). On the basis of the patient’s clinical manifestations, pathological findings, mNGS results, and response to pharmacotherapy, a diagnosis of co-infection with *E. orientalis* and *M. fortuitum* was considered. The high susceptibility of *M. fortuitum* to omadacycline *in vitro* was considered here (Rizzo and Moniri, 2022). The multidisciplinary team (MDT) discussion recommended an anti-infective regimen, including levofloxacin 0.5 g orally once daily, isavuconazole 200 mg via intravenous infusion once daily, omadacycline 100 mg via intravenous infusion once daily, and amikacin 0.4 g via intravenous infusion every 12 h. This recommendation aligns with the literature on the treatment of *E. orientalis* (Reddy et al., 2023) and the 2020 IDSA guidelines (Daley et al., 2020) for the treatment of NTM infections. Although the fever curve came down, it still remained above normal. The anti-infective regimen was adjusted to 0.5 g of levofloxacin orally once daily, 200 mg of isavuconazole via intravenous infusion once daily, and 100 mg of minocycline orally twice a day, plus 0.4 g of amikacin via intravenous infusion every 12 h. Within 48 h, the temperature was maintained at 37°C. Encouraged, we peeled off amikacin first, then minocycline—only to watch a low-grade fever creep back. Reinstating both drugs brought the temperature back in line. After 1 month, the follow-up CT showed a marked reduction in the lesion, which measured approximately 1.8 cm × 1.7 cm (**Figure 3B**). At this point, we discontinued amikacin and kept the patient on clarithromycin 0.5 g orally once daily, levofloxacin 0.5 g orally once daily, isavuconazole 200 mg orally once daily, and minocycline 100 mg orally twice a day. After another 4 weeks on this leaner regimen, the mediastinal mass had melted down to a 1.3-cm × 1.1-cm nub (**Figure 3C**). Therefore, minocycline was withdrawn, and treatment with clarithromycin 0.5 g orally once daily, levofloxacin 0.5 g orally once daily, and isavuconazole 200 mg orally once daily was continued. The patient felt well and underwent treatment for the condition.

## Discussion and conclusion

*E. orientalis* is a dimorphic fungus belonging to the emerging fungal genus *Emergomyces* (ES), which is widely distributed in soil and has been identified in Africa, North America, Europe, and Asia. On the basis of its geographical distribution, it is classified as *Emergomyces pasteurianus*, *Emergomyces africanus*, *Emergomyces canadensis*, *Emergomyces europaeus*, or *E. orientalis* (Schwartz et al., 2019). According to the literature, *Emergomyces* infections are more common in immunocompromised populations; however, immunocompetent individuals can also be affected (Heys et al., 2014; Govender and Grayson, 2019; Vinayagamoorthy et al., 2023; Jiang et al., 2024). Five cases of *E. orientalis* infection have been reported in China, two of which occurred in immunocompetent patients. The case described in this study involved an immunocompetent patient with mediastinal infection. The patient frequently worked outdoors and was exposed to significant dust inhalation during activities such as training, running, and mountaineering, which may have been important predisposing factors for *E. orientalis* infection. Based on the patient’s clinical presentation, chest CT findings, histopathological evidence of yeast-like fungi, mNGS results, and response to treatment, a diagnosis of a mediastinal co-infection of *E. orientalis* and NTM was made.

*Emmonsia* and NTM infections can involve multiple organs and commonly affect the lungs, lymph nodes, skin, subcutaneous tissues, and bones (Schwartz et al., 2015; Chin et al., 2020; Pierce et al., 2023). The present case presented with isolated mediastinal lymphadenopathy, which lacked specific clinical manifestations and was difficult to distinguish from other common conditions, such as tuberculosis, lymphoma, and sarcoidosis, thus easily leading to misdiagnosis. Currently, the diagnosis of *E. orientalis* and *M. fortuitum* relies primarily on the patient’s immune status, clinical presentation, culture results, histopathology, and molecular diagnostic techniques, including PCR and mNGS (Daley et al., 2020; Thompson et al., 2021). In this case, the patient was immunocompetent with atypical clinical features, and sampling of the mediastinal lesion was challenging. Both pathogens were difficult to detect using conventional methods. Although yeast-like fungi were observed on both GMS and PAS staining, species identification was not possible. *M. fortuitum*, as a rapidly growing NTM, has a low positivity rate in conventional culture and must be differentiated from other NTM and *M. tuberculosis*. In contrast, mNGS enables comprehensive and efficient sequencing of pathogenic microorganisms and is currently recommended by laboratory and pharmaceutical experts for species identification of *E. orientalis* (Schwartz et al., 2019; He et al., 2021). In this case, two EBUS-guided biopsies of the lesion provided high-quality specimens for histopathological and pathogenetic analyses, which were critical in achieving a definitive diagnosis. In addition, mNGS of the lesion tissue rapidly identified two rare pathogens, providing key evidence for diagnosis and guiding treatment adjustment. This suggests that, for patients with unexplained mediastinal masses clinically suspected of unusual pathogen infection, EBUS combined with mNGS can be considered as a diagnostic strategy. However, it is important to emphasize that interpretation of the mNGS results requires careful consideration of multiple factors, such as the microbiological characteristics and clinical presentation, among others.

In this case, *M. fortuitum* was detected with mNGS in the first stage, but was not considered the primary pathogen at the time for the following reasons:

1. The sequence reads for *M. fortuitum* were low, and there was no typical radiological presentation of NTM infection.

2. The virulence of *M. fortuitum* is limited, and its clinical significance when detected in respiratory specimens has been reported to be low in the literature (Simons et al., 2011).

3. *M. fortuitum* is a classic waterborne contaminant in hospital settings. False-positive results could not be excluded due to potential contamination of the water sources used during tissue processing (e.g., rinsing and dehydration).

4. Prior to the initiation of liposomal amphotericin B, the patient received empirical therapy with imipenem cilastatin, which was ineffective. The fever persisted, and follow-up chest CT showed no reduction in the mediastinal lesions.

Given the concurrent detection of *E. orientalis*, a pathogenic dimorphic fungus with a relatively high virulence, and the fact that NTM treatment typically requires prolonged, multidrug antimicrobial regimens, *M. fortuitum* was not considered an active pathogen in the first stage, and no specific anti-NTM therapy was initiated. In the second stage, compared with the first stage, *M. fortuitum* was again detected by mNGS, with significantly more sequence reads. Given the poor response to antifungal therapy alone, *M. fortuitum* was considered a contributing pathogen in the second stage. While *Legionella* sequences were detected in the pathological tissue by mNGS, their clinical significance in this case was difficult to determine definitively. *Legionella* is a ubiquitous environmental microorganism commonly found in water and soil, and its detection in tissue samples can, in some cases, result from environmental contamination during specimen processing. Furthermore, the patient’s clinical presentation lacked several features typical of *Legionella* infection, such as high-grade fever, significant extrapulmonary manifestations (e.g., hyponatremia), and classic radiological findings of patchy or lobar consolidation. The treatment regimen, which included agents active against *Legionella*, adds another layer of complexity to the interpretation. On balance, we considered *E. orientalis* and *M. fortuitum* as the most likely primary pathogens, and while *Legionella* could not be definitively excluded, it was judged less likely to be the main driver of disease in the absence of supportive clinical, radiological, or environmental evidence.

Currently, there are no global guideline recommendations for the treatment of *E. orientalis* infection. According to literature reports on *E. orientalis* infection, the main therapeutic agents are amphotericin B and azoles (Wang et al., 2017; Luo et al., 2024). Therefore, in the initial stage of this case, liposomal amphotericin B was used for induction therapy, followed by sequential voriconazole therapy after lesion reduction. However, the patient subsequently experienced disease recurrence. Repeated mNGS suggested co-infection with *M. fortuitum*.

For *M. fortuitum*, combination regimens, such as macrolides, quinolones, and aminoglycosides, should be selected on the basis of drug susceptibility testing (Daley et al., 2020). In this case, drug susceptibility testing for *M. fortuitum* was not feasible. Based on multidisciplinary expert consultation and the literature, the anti-NTM regimen initially consisted of levofloxacin, omadacycline, and amikacin. Considering potential drug–drug interactions, the antifungal agent was switched to isavuconazole. Although the patient’s peak body temperature decreased, low-grade fever persisted. In accordance with the IDSA guidelines for the diagnosis and treatment of NTM infections, the anti-infective regimen was adjusted in the second stage to include the four-drug combination of isavuconazole, levofloxacin, minocycline, and amikacin. The regimen was later optimized to an oral maintenance therapy based on efficacy and drug tolerance.

Throughout the treatment course, close attention was paid to drug interactions and adverse effects: the nephrotoxicity of liposomal amphotericin B, the hepatotoxicity risk of azoles combined with NTM drugs, and the ototoxicity/nephrotoxicity of aminoglycosides. Regular monitoring of liver and kidney function and dose adjustments are required to balance efficacy and safety. A literature review indicates that the treatment response in patients with *E. orientalis* infection is closely related to the immune status (Vinayagamoorthy et al., 2023): immunocompetent patients may require shorter treatment durations, while co-infection with NTM necessitates longer treatment courses and a more intensive combination therapy. This patient was immunocompetent, and the treatment duration was individualized accordingly. This case highlights that, in patients with unexplained fever, mediastinal masses, and poor response to conventional anti-infective therapy, the possibility of rare pathogens and co-infections should be considered.

This study has several limitations. Firstly, it is a retrospective observational study. Secondly, the sample size is small. Thirdly, there are no established guidelines for co-infection with *E. orientalis* and *M. fortuitum*. The treatment regimen was individualized and exploratory, and the optimal drug combination, duration, and criteria for discontinuation require further validation.

## Data Availability

The original contributions presented in the study are included in the article/**Supplementary Material**. Further inquiries can be directed to the corresponding authors.

## Supplementary material

Supplementary Figure 1 Changes in positron emission tomography computed tomography (PET-CT) images of the patient. **(A, B)** Baseline PET-CT demonstrates enlargement of the 2R and 4R lymph node stations, measuring 3.06 cm× 2.19 cm, with a maximum standardized uptake value (SUVmax) of 6.56. **(C, D)** PET-CT performed two weeks after discharge from the first phase of treatment shows enlargement of the mediastinal lesions. The lesions demonstrate persistent and slightly increased metabolic activity, with an SUVmax of 6.72.
