# Supplementary figures and images for: Diagnosis and treatment of a patient with mediastinal infection caused by Emergomyces orientalis and Mycobacterium fortuitum
Source: Front Cell Infect Microbiol. 2026 Apr 24;16:1778930. doi: 10.3389/fcimb.2026.1778930 (PMC13152838; doi:10.3389/fcimb.2026.1778930)

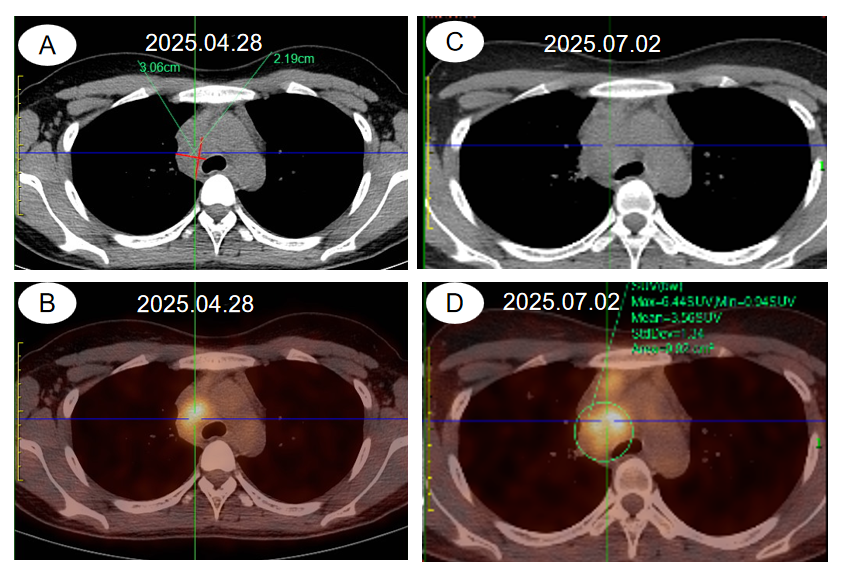

Supplement: Supplementary Figure 1 — Changes in positron emission tomography computed tomography (PET-CT) images of the patient. (A, B) Baseline PET-CT demonstrates enlargement of the 2R and 4R lymph node stations, measuring 3.06 cm× 2.19 cm, with a maximum standardized uptake value (SUVmax) of 6.56. (C, D) PET-CT performed two weeks after discharge from the first phase of treatment shows enlargement of the mediastinal lesions. The lesions demonstrate persistent and slightly increased metabolic activity, with an SUVmax of 6.72. [file Image1.png]
